# Pertuzumab, trastuzumab, and docetaxel for Chinese patients with previously untreated HER2-positive locally recurrent or metastatic breast cancer (PUFFIN): a phase III, randomized, double-blind, placebo-controlled study

**DOI:** 10.1007/s10549-020-05728-w

**Published:** 2020-06-20

**Authors:** Binghe Xu, Wei Li, Qingyuan Zhang, Zhimin Shao, Qiao Li, Xiaojia Wang, Huiping Li, Tao Sun, Yongmei Yin, Hong Zheng, Jifeng Feng, Hong Zhang, Guiyuan Lei, Eleonora Restuccia

**Affiliations:** 1 National Cancer Center/National Clinical Research Center for Cancer/Cancer Hospital, Chinese Academy of Medical Sciences, and Peking Union Medical College, Beijing, China; 2 The Cancer Center, The First Hospital of Jilin University, Jilin, China; 3 Harbin Medical University, Harbin, China; 4 Fudan University Shanghai Cancer Center, Shanghai, China; 5 Zhejiang Cancer Hospital, Hangzhou City, China; 6 Beijing Cancer Hospital, Beijing, China; 7 Cancer Hospital of China Medical University, Liaoning Cancer Hospital & Institute, Liaoning, China; 8 Jiangsu Province Hospital, Nanjing, China; 9 West China Hospital, Sichuan University, Chengdu, China; 10 Jiangsu Cancer Hospital, Nanjing, China; 11 Roche (China) Holding Ltd, Shanghai, China; 12 Roche Products Limited, Welwyn Garden City, UK; 13 F. Hoffmann-La Roche Ltd, Basel, Switzerland; 14 Present Address: Janssen China R&D, Beijing, China

**Keywords:** Pertuzumab, Metastatic breast cancer, Locally recurrent breast cancer, HER2, Chinese

## Abstract

**Purpose:**

The Chinese bridging study PUFFIN (NCT02896855) aimed to assess consistency of efficacy with CLEOPATRA (NCT00567190), investigating pertuzumab with trastuzumab and docetaxel versus placebo, trastuzumab, and docetaxel in patients with previously untreated HER2-positive locally recurrent or metastatic breast cancer.

**Methods:**

Patients were randomized 1:1, stratified by visceral/non-visceral disease and hormone receptor status. The primary endpoint was investigator-assessed progression-free survival (PFS). Secondary endpoints included objective response rate (in patients with measurable baseline disease), overall survival, and safety. The consistency threshold for PFS (hazard ratio [HR] < 0.81) (maintaining ≥ 50% of the risk reduction determined in CLEOPATRA [HR 0.62]) determined the target sample size (*n* = 240).

**Results:**

Two hundred forty-three patients were randomized. Median PFS was 14.5 months in the pertuzumab arm (95% confidence interval [CI] 12.5, 18.6) and 12.4 months in the placebo arm (95% CI 10.4, 12.7) in the intention-to-treat population (HR: 0.69 [95% CI 0.49, 0.99]). Objective responses were recorded in 83/105 (79.0%) and 67/97 (69.1%) patients, respectively. Grade ≥ 3 adverse events (70.5% and 69.2%, respectively) and serious adverse events (19.7% and 19.2%, respectively) were similar across both arms. No heart failure cases or symptomatic left ventricular ejection fraction declines were reported.

**Conclusions:**

PUFFIN met its primary objective. Overall, efficacy data were consistent with CLEOPATRA. Safety was consistent with the known pertuzumab safety profile. PUFFIN adds to the totality of data with pertuzumab in previously untreated HER2-positive locally recurrent or metastatic breast cancer and supports the favorable benefit–risk profile of pertuzumab in Chinese patients

**Trial registration:**

ClinicalTrials.gov, NCT02896855, registered 7 September 2016

**Electronic supplementary material:**

The online version of this article (10.1007/s10549-020-05728-w) contains supplementary material, which is available to authorized users.

## Introduction

The anti-human epidermal growth factor receptor 2 (HER2) antibodies pertuzumab and trastuzumab (PERJETA^®^ and Herceptin^®^; F. Hoffmann-La Roche Ltd, Basel, Switzerland) have complementary modes of antitumor activity as a result of their binding to different domains on the HER2 protein [1, 2].

In the CLEOPATRA study (NCT00567190), adding pertuzumab to trastuzumab and docetaxel significantly improved progression-free and overall survival (PFS and OS) compared with placebo, trastuzumab, and docetaxel in patients with previously untreated HER2-positive locally recurrent or metastatic breast cancer [3–5]. The median PFS was 18.5 months in the pertuzumab arm and 12.4 months in the placebo arm (hazard ratio [HR] 0.62 [95% confidence interval (CI) 0.51, 0.75]) at the primary analysis [3] and the OS benefit resulted in a significant 34% reduction in risk of death (HR 0.66; 95% CI 0.52, 0.84; *p* = 0.0008) at the confirmatory OS analysis after an additional year of follow-up [4]. In an exploratory analysis [6], patients from Asia (China including Hong Kong, plus Japan, Korea, the Philippines, Singapore, and Thailand) experienced more adverse events, especially febrile neutropenia (which may have been related to increased diarrhea and mucosal inflammation), than patients from Europe, North America, and South America combined, leading to a higher proportion of docetaxel dose reductions in patients from Asia. Despite these adjustments, comparable survival benefits across regions were suggested.

PUFFIN (NCT02896855) is a bridging study that aims prospectively to assess the efficacy of pertuzumab, trastuzumab, and docetaxel in Chinese patients, and determine consistency with the benefit observed in the global population of CLEOPATRA.

## Methods

PUFFIN is a phase III, randomized, double-blind, placebo-controlled study conducted across 15 centers in China. The protocol (available online) was approved by the institutional review board at each participating site. All participants provided written informed consent.

Patients had HER2-positive (centrally confirmed immunohistochemistry [IHC] 3 + or in situ hybridization-positive) locally recurrent or metastatic breast cancer, no prior therapy for metastatic disease (except for one hormonal regimen before randomization, if indicated; hormone receptor status was also centrally confirmed), no prior tyrosine kinase inhibitors or anti-HER2 therapies (except for trastuzumab in the neoadjuvant or adjuvant settings, if indicated), and a disease-free interval of 12 months or more. Patients were eligible if they were 18 years of age or older, had measurable or non-measurable disease, a left ventricular ejection fraction (LVEF) of 55% or more at baseline (via echocardiography or multiple-gated acquisition scan), and an Eastern Cooperative Oncology Group performance status (ECOG PS) of 0 or 1. Patients were excluded if they had received prior treatment for metastatic breast cancer (with the above caveats), prior doxorubicin (or equivalent anthracycline) exposure of 360 mg/m^2^ or above, had a history of LVEF decline to less than 50% during or after trastuzumab in the neoadjuvant or adjuvant settings, or if they had any other conditions that were not controlled and could affect the patient’s ability to comply with the study. Treatment with other systemic anticancer agents (e.g., chemotherapy, hormonal therapy, immunotherapy) or non-protocol-specified anticancer therapies was not allowed.

### Procedures

Eligible patients were randomized (permuted block scheme via an interactive voice- or web-based response system; block size four; sequence generated by PAREXEL [Waltham, MA]) 1:1 to pertuzumab (840 mg loading dose, followed by 420 mg every 3 weeks), trastuzumab (8 mg/kg loading dose, then 6 mg/kg every 3 weeks), and docetaxel (75 mg/m^2^ every 3 weeks) or placebo, trastuzumab, and docetaxel. Randomization was stratified by visceral versus non-visceral disease and hormone receptor status (estrogen and progesterone receptor-negative vs. estrogen and/or progesterone receptor-positive). Pertuzumab and trastuzumab were given until disease progression or unacceptable toxicity. After completion of Cycle 6, discontinuation of docetaxel treatment was at the discretion of the patient and treating physician. Dose reductions were not permitted for placebo, pertuzumab, or trastuzumab. Docetaxel doses could be reduced to 55 mg/m^2^ if febrile neutropenia or severe or cumulative cutaneous reactions occurred. All study drugs were given intravenously.

### Assessments

The primary endpoint was investigator-assessed PFS, defined as the time from randomization to the first occurrence of disease progression (assessed per Response Evaluation Criteria In Solid Tumors version 1.1 [RECIST v1.1]) [7]; or death from any cause within 18 weeks after the last tumor assessment. Secondary endpoints included investigator-assessed objective response rate in patients with measurable baseline disease (defined as a complete or partial response, assessed per RECIST v1.1), OS, and safety.

Tumor assessments were conducted every 9 weeks from randomization until progression. LVEF assessments were performed at screening, baseline, every 9 weeks from randomization until the treatment discontinuation visit (or more frequently as needed), every 6 months thereafter for the first year, and annually for up to 3 years until the end of the study. Laboratory tests were carried out and ECOG PS assessed at each cycle and at the treatment discontinuation visit. Adverse events were continuously monitored throughout the study and were graded per the National Cancer Institute’s Common Terminology Criteria for Adverse Events, version 4.0. Data for specific events to monitor (i.e., those associated with potential risks or those that had missing information) are also presented.

### Statistical methods

The target sample size of 240 patients was determined based on the consistency threshold for PFS, defined as a HR < 0.81, which maintains ≥ 50% of the risk reduction determined in CLEOPATRA (HR 0.62). One hundred twenty-three events in the two treatment arms were required to provide an appropriate 83% probability of showing consistency. The sample size estimation was based on the following assumptions: the median PFS was 11 months in the control arm, with a PFS HR of 0.68, and the recruitment period was estimated to be 15 months.

The Kaplan–Meier approach was used to estimate median PFS for each treatment arm. The Cox proportional hazards model, stratified by disease type (visceral vs. non-visceral disease) and hormone receptor status, was used to estimate the HR and its 95% CI. The two-sided stratified log-rank test was used to compare PFS between the two treatment arms. An estimate of the objective response rate and its 95% CI (Clopper–Pearson) were calculated for each treatment arm. The difference in objective response was also calculated with 95% CIs (Hauck–Anderson).

Statistical testing is considered exploratory. Safety analyses are descriptive.

## Results

### Study population

During the period from 13 September 2016 to 28 September 2017, a total of 243 female patients were randomized; 122 to the pertuzumab arm and 121 to the placebo arm (intention-to-treat populations; Online Resource 1: Fig. S1). One patient randomized to the placebo arm discontinued from the study before receiving any study drug. The safety populations therefore comprised 122 and 120 patients in the pertuzumab and placebo arms, respectively. The clinical cut-off date was 27 June 2018. Median follow-up was 13.7 months in the pertuzumab arm and 13.1 months in the placebo arm. The baseline demographics and disease characteristics were generally balanced between arms (Table 1). At baseline, the majority of patients enrolled had visceral disease (71.6%), and 56.8% had ECOG PS of 1. The percentage of patients with hormone receptor-positive status was 58.4%, and the proportion of patients with lower HER2 expression (HER2 IHC 2 +) was 25.9%. Prior adjuvant or neoadjuvant therapy had been given in 66.7% of enrolled patients, with prior trastuzumab being given in 11.1%. In terms of concomitant medications typically used to manage toxicity of chemotherapy in the pertuzumab arm, 95.9% of patients received steroids, 86.9% received 5-HT3 antagonists, and 78.7% received colony stimulating factors.

**Table 1:** Baseline patient demographics and disease characteristics for the intention-to-treat population

Characteristic	Pertuzumab plus trastuzumab plus docetaxel (*n* = 122)	Placebo plus trastuzumab plus docetaxel (*n* = 121)
Female sex, *n* (%)	122 (100)	121 (100)
Age, years		
Median	51.0	53.0
Range	26–74	25–71
ECOG PS, *n* (%)		
0	56 (45.9)	49 (40.5)
1	66 (54.1)	72 (59.5)
Disease type at screening, *n* (%)		
Non-visceral	34 (27.9)	35 (28.9)
Visceral	88 (72.1)	86 (71.1)
Hormone receptor status, *n* (%)		
ER-positive, PgR-positive, or both	69 (56.6)	73 (60.3)
ER-negative and PgR-negative	53 (43.4)	48 (39.7)
*HER2* status, assessed by IHC^a^, *n* (%)		
1+	1 (0.8)	3 (2.5)
2+	34 (28.8)	29 (24.2)
3+	83 (70.3)	88 (73.3)
*HER2* status, assessed by FISH, *n* (%)		
Positive	119 (97.5)	120 (100)
Negative	3 (2.5)	0 (0)
Prior adjuvant or neoadjuvant therapy		
No	46 (37.7)	35 (28.9)
Yes	76 (62.3)	86 (71.1)
Hormonal	30 (24.6)	37 (30.6)
Trastuzumab	17 (13.9)	10 (8.3)

*ECOG PS* Eastern Cooperative Oncology Group performance status, *ER* estrogen receptor, *FISH* fluorescence in situ hybridization, *HER2* human epidermal growth factor receptor 2, *IHC* immunohistochemistry, *PgR* progesterone receptor

^a^*n* = 118 in the pertuzumab arm and *n* = 120 in the placebo arm

### Progression-free survival

**Fig. 1:**
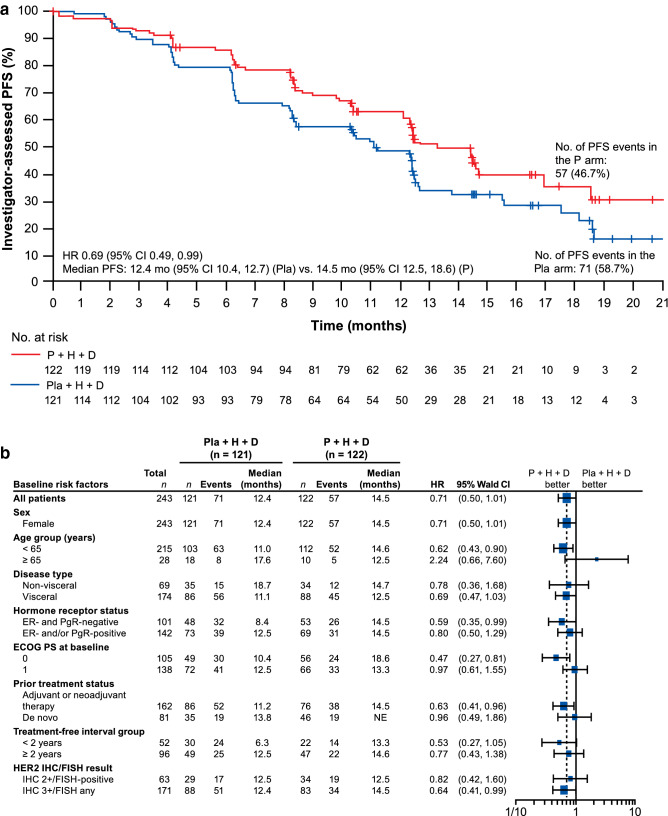
Investigator-assessed PFS in **a** the intention-to-treat population and **b** subgroups. *CI* confidence interval, *D* docetaxel, *ECOG PS* Eastern Cooperative Oncology Group performance status, *ER* estrogen receptor, *FISH* fluorescence in situ hybridization, *H* trastuzumab, *HER2* human epidermal growth factor receptor 2, *HR* hazard ratio, *IHC* immunohistochemistry, *P* pertuzumab, *PFS*, progression-free survival, *PgR* progesterone receptor, *Pla* placebo

Treatment with pertuzumab plus trastuzumab plus docetaxel resulted in an improvement in investigator-assessed PFS compared with placebo plus trastuzumab plus docetaxel. The median PFS was 14.5 months in the pertuzumab arm (95% CI 12.5, 18.6) compared with 12.4 months in the placebo arm (95% CI 10.4, 12.7). The HR was 0.69 (95% CI 0.49, 0.99) (Fig. 1a). PFS events occurred in 57 patients in the pertuzumab arm (46.7%) and 71 patients in the placebo arm (58.7%). Subgroup analyses were consistent with overall PFS results (Fig. 1b); there were 7 and 11 patients with brain metastases in the pertuzumab and placebo arms, respectively.

### Key secondary efficacy endpoints

Patients with measurable disease at baseline in the pertuzumab arm achieved an objective response rate of 79.0% compared with 69.1% in the placebo arm; a difference of 9.98% (95% CI − 2.65%, 22.60%) (Table 2; an exploratory analysis by hormone receptor subgroups is shown in Online Resource 2: Table S1).

**Table 2:** Objective response rate in patients with measurable disease at baseline

Response	Pertuzumab plus trastuzumab plus docetaxel (*n* = 105)	Placebo plus trastuzumab plus docetaxel (*n* = 97)
Objective response, *n* (%)	83 (79.0)	67 (69.1)
Difference	9.98 (95% CI − 2.65, 22.60^a^) *p* = 0.1126	
Complete response, *n* (%)	6 (5.7)	8 (8.2)
Partial response, *n* (%)	77 (73.3)	59 (60.8)
Stable disease, *n* (%)	16 (15.2)	20 (20.6)
Progressive disease, *n* (%)	4 (3.8)	4 (4.1)
Missing or unavailable, *n* (%)	2 (1.9)	6 (6.2)

*CI* confidence interval

^a^Hauck–Anderson CI

Only 25 deaths were reported at the time of clinical cut-off (13 [10.7%] in the placebo arm and 12 [9.8%] in the pertuzumab arm). The median time to death had not been reached in either treatment arm.

### Treatment exposure

The median number of pertuzumab or placebo cycles received by patients in the safety population was 18.0 in the pertuzumab arm (range 1–31) and 15.5 in the placebo arm (1–32). Patients received pertuzumab or placebo treatment for median durations of 54.2 weeks (range 3–93 weeks) and 47.8 weeks (range 3–96 weeks), respectively.

Patients in the pertuzumab arm received a median of 7.0 docetaxel cycles (range 1–21) and patients in the placebo arm received a median of 6.5 (range 1–22). Patients received docetaxel with a median total dose of 922.0 mg in the pertuzumab arm and 826.1 mg in the placebo arm. Reasons for permanent discontinuation of all study treatments are described in Online Resource 1: Fig. S1. Disease progression was the most common reason for discontinuation of all study treatments.

### Safety

The safety profile during the treatment period is shown in Table 3. Data for specific events of interest to pertuzumab therapy, including events to monitor (i.e., those that the health authorities requested to be monitored closely; usually potential risks for missing information), are presented in Online Resource 3: Table S2. Of the most common adverse events (occurring in ≥ 10% of patients and with a difference of ≥ 5% between arms), any-grade anemia, alopecia, diarrhea, pyrexia, cough, hypokalemia, and stomatitis were higher in the pertuzumab arm. Conversely, any-grade increased alanine aminotransferase, increased aspartate aminotransferase, peripheral edema, and increased weight were higher in the placebo arm. Grade ≥ 3 adverse events and serious adverse events were similar across arms (grade ≥ 3 events; 70.5% in the pertuzumab arm vs. 69.2% in the placebo arm, serious events; 19.7% in the pertuzumab arm vs. 19.2% in the placebo arm). Neutropenia, leukopenia, febrile neutropenia, diarrhea, and anemia were the most common grade ≥ 3 adverse events in both arms (≥ 3%). Leukopenia and anemia showed higher incidences (≥ 2%) in the pertuzumab arm compared with the placebo arm, while neutropenia was higher in the placebo arm.

**Table 3:** Safety summary in the safety population

Patients with at least one event:	Pertuzumab plus trastuzumab plus docetaxel (*n* = 122)	Placebo plus trastuzumab plus docetaxel (*n* = 120)
Most common adverse events (all grades)^a^, *n* (%)		
Leukopenia	89 (73.0)	86 (71.7)
Neutropenia	86 (70.5)	84 (70.0)
Anemia	64 (52.5)	57 (47.5)
Alopecia	50 (41.0)	40 (33.3)
Alanine aminotransferase increased	35 (28.7)	50 (41.7)
Diarrhea	56 (45.9)	26 (21.7)
Aspartate aminotransferase increased	33 (27.0)	42 (35.0)
Asthenia	25 (20.5)	20 (16.7)
Pyrexia	26 (21.3)	18 (15.0)
Pain	18 (14.8)	22 (18.3)
Cough	23 (18.9)	14 (11.7)
Decreased appetite	15 (12.3)	14 (11.7)
Peripheral edema	10 (8.2)	18 (15.0)
Nail discoloration	12 (9.8)	15 (12.5)
Nausea	13 (10.7)	14 (11.7)
Upper respiratory tract infection	12 (9.8)	13 (10.8)
Blood bilirubin increased	10 (8.2)	13 (10.8)
Hypokalemia	15 (12.3)	7 (5.8)
Vomiting	13 (10.7)	9 (7.5)
Hypoesthesia	13 (10.7)	8 (6.7)
Weight increased	4 (3.3)	15 (12.5)
Stomatitis	14 (11.5)	4 (3.3)
Grade 3 or higher adverse events^b^, *n* (%)		
Neutropenia	67 (54.9)	70 (58.3)
Leukopenia	60 (49.2)	56 (46.7)
Febrile neutropenia	5 (4.1)	7 (5.8)
Diarrhea	5 (4.1)	3 (2.5)
Anemia	6 (4.9)	0 (0)

Table includes adverse events with onset from first dose of study drug through 42 days after last dose of study drug

^a^Reported in ≥ 10% of patients in either arm

^b^Reported in ≥ 3% of patients in either arm

Adverse events or death led to the withdrawal of six patients (4.9%) from pertuzumab treatment and two (1.7%) from placebo treatment. In the pertuzumab arm, three patients were withdrawn due to adverse events (2.5%; two due to decreased ejection fraction, one due to pneumonia at Cycle 4) and three were withdrawn due to death. Of the three deaths in the pertuzumab arm, two were cardiovascular related (cardiac tamponade, which occurred in patients who had a known cardiovascular history early on in the study), and one was due to general disorder.

No cases of heart failure or symptomatic LVEF decline were reported. Two patients (1.7%) in the pertuzumab arm and none in the placebo arm experienced LVEF decreases of ≥ 10 percentage points from baseline and to an absolute value of < 50% (one of these patients experienced a decrease of ≥ 15 percentage points). One patient recovered with drug discontinuation and one recovered without.

Upon cessation of chemotherapy, when patients received HER2-targeted therapy only, the incidence of adverse events of interest to pertuzumab treatment declined: leukopenia was reported in 88/122 patients in the pertuzumab arm (72.1%) and 86/120 patients in the placebo arm (71.7%) during the chemotherapy treatment period, compared with 15/98 patients in the pertuzumab arm (15.3%) and 15/86 patients in the placebo arm (17.4%) following discontinuation or completion of docetaxel. Grade ≥ 3 diarrhea was experienced by five patients in the pertuzumab arm (4.1%) and two patients in the placebo arm (1.7%) during the chemotherapy treatment period, compared with no patients in the pertuzumab arm and one patient in the placebo arm (1.2%) during the chemotherapy-free period.

In the safety population, the majority of deaths were attributable to disease progression: nine (7.4%) in the pertuzumab arm and 10 (8.3%) in the placebo arm. The number of fatal adverse events was similar across both groups (three [2.5%] in the pertuzumab and two [1.7%] in the placebo arm). In the pertuzumab arm, two patients died due to cardiac tamponade, and one death was ‘unexplained’, while in the placebo arm, one death was due to respiratory failure and one was ‘unexplained’.

## Discussion

The PUFFIN study met its primary objective and, overall, efficacy data demonstrated consistency with the results of the CLEOPATRA study [3]. Pertuzumab plus trastuzumab plus docetaxel prolonged PFS in Chinese patients with HER2-positive locally recurrent or metastatic breast cancer, with a clinically meaningful benefit corresponding to a 31% reduction in the risk of disease progression or death (HR 0.69 [95% CI 0.49, 0.99]). The treatment effect was consistent throughout most of the patient subgroups analyzed. In the global study, CLEOPATRA, the HR for the statistically meaningful improvement of PFS with the addition of pertuzumab to trastuzumab and docetaxel was 0.62 (95% CI 0.51, 0.75).

In PUFFIN, favorable efficacy with pertuzumab was also observed in terms of response rate, a secondary objective of the study, which was higher in the pertuzumab arm compared with the placebo arm (79.0% and 69.1%, respectively). This was also similar to what was observed in CLEOPATRA (80.2% in the pertuzumab arm and 69.3% in the placebo arm [3]).

It should be noted that differences exist between CLEOPATRA and PUFFIN. Specifically, in terms of study design, PFS was investigator—assessed per RECIST v1.1 in PUFFIN, whereas in CLEOPATRA, PFS was independent review facility—assessed per RECIST v1.0. This was in accordance with acceptable study requirements for bridging studies in China and reflected updates to international RECIST guidelines at that time. Importantly, in the absence of an independent review facility, the randomized, double-blind design of PUFFIN already provided a sound mitigation strategy for the risk of bias.

PUFFIN was a bridging study, and as such, was not designed to show superiority of the pertuzumab arm over the placebo arm in terms of OS. At the clinical cut-off date for the primary analysis, very few OS events had occurred, with 25 deaths reported in total. OS data for PUFFIN will be updated for the final clinical study report. In addition, the point estimate for median PFS in the pertuzumab arm remains relatively immature due to the small number of events and the fact that the median follow-up in this arm was shorter than the median PFS value.

The combination of pertuzumab, trastuzumab, and docetaxel was tolerable in the Chinese population. The overall safety profile was consistent with that observed in CLEOPATRA in the metastatic setting, and generally in line with the known pertuzumab safety profile, with no new or unexpected signals reported. In particular, the addition of pertuzumab to trastuzumab and docetaxel did not increase the incidence of grade ≥ 3 adverse events and serious adverse events in the Chinese population. Neutropenia, leukopenia, febrile neutropenia, diarrhea, and anemia were the most common grade ≥ 3 adverse events in both arms.

As would be expected based on previous experience with pertuzumab in the early and metastatic settings [3, 8–10], the incidence of adverse events of interest to pertuzumab treatment decreased upon cessation of chemotherapy; leukopenia and grade ≥ 3 diarrhea both occurred more frequently in the chemotherapy period versus the chemotherapy-free period.

Despite a higher rate of patients discontinuing therapy due to adverse events in the pertuzumab arm, treatment discontinuation or interruptions due to adverse events were lower overall in the PUFFIN study in both treatment arms compared with CLEOPATRA.

In PUFFIN, the pattern of adverse events and the safety profile of the pertuzumab combination were similar to those seen in CLEOPATRA, but there were fewer adverse events leading to discontinuations, possibly indicating that in PUFFIN the investigators were able to better manage toxicities; as such, reducing the need to discontinue treatment.

Among the concomitant medications typically used to manage toxicities of chemotherapy, there was high use of growth factors (in 79% of the patients) during treatment in the pertuzumab arm in PUFFIN. In CLEOPATRA, growth factors were administered to manage toxicity in 28% of patients in the pertuzumab arm [4]. Steroids (96%) and 5-HT3 antagonists (87%), usually given to prevent nausea and vomiting post-chemotherapy administration, were also used frequently in PUFFIN. This could reflect both better management of the pertuzumab-containing regimen over time, or regional differences in standard practice and adoption of toxicity management approaches versus the global population. LVEF decreases were less common in PUFFIN than in CLEOPATRA (1.7% of patients in the pertuzumab arm of PUFFIN [none in the placebo arm] vs. 3.8% and 6.6%, respectively, in CLEOPATRA) [3]. Importantly, as in CLEOPATRA, no cases of heart failure or symptomatic LVEF decrease were reported in PUFFIN.

## Conclusions

In summary, PUFFIN is the first randomized, placebo-controlled, phase III study of trastuzumab and docetaxel in combination with pertuzumab or placebo in Chinese patients with previously untreated HER2-positive locally recurrent or metastatic breast cancer. These results reinforce the existing large body of evidence for pertuzumab in HER2-positive breast cancer, and support the favorable benefit–risk profile of the pertuzumab-based regimen in Chinese patients in the first-line metastatic setting.

## Electronic supplementary material

Below is the link to the electronic supplementary material.

Supplementary file1 (DOCX 189 kb)

## Data Availability

Qualified researchers may request access to individual patient-level data through the clinical study data request platform: https://vivli.org/. Further details on Roche's criteria for eligible studies are available here: https://vivli.org/members/ourmembers/. For further details on Roche's Global Policy on the Sharing of Clinical Information and how to request access to related clinical study documents, see here: https://www.roche.com/research_and_development/who_we_are_how_we_work/clinical_trials/our_commitment_to_data_sharing.htm.

## Abbreviations

CI: Confidence interval
D: Docetaxel
ECOG PS: Eastern Cooperative Oncology Group performance status
ER: Estrogen receptor
FISH: Fluorescence in situ hybridization
H: Trastuzumab
HER2: Human epidermal growth factor receptor
HR: Hazard ratio
ICH: International Council on Harmonisation
IEC: Independent ethics committee
IHC: Immunohistochemistry
LVEF: Left ventricular ejection fraction
OS: Overall survival
PFS: Progression-free survival
PgR: Progesterone receptor
Pla: Placebo
RECIST: Response Evaluation Criteria In Solid Tumors
