# Supplementary material for: Pertuzumab, trastuzumab, and docetaxel for Chinese patients with previously untreated HER2-positive locally recurrent or metastatic breast cancer (PUFFIN): a phase III, randomized, double-blind, placebo-controlled study
Source: Breast Cancer Res Treat. 2020 Jun 20;182(3):689–97. doi: 10.1007/s10549-020-05728-w (PMC7320929; doi:10.1007/s10549-020-05728-w)
Supplement: Supplementary file 1 — Supplementary file1 (DOCX 189 kb) [file 10549_2020_5728_MOESM1_ESM.docx]

**Journal:** Breast Cancer Research and Treatment

**Article type:** Clinical Trial

**Pertuzumab, trastuzumab, and docetaxel for Chinese patients with previously untreated HER2-positive locally recurrent or metastatic breast cancer (PUFFIN): a phase III, randomized, double-blind, placebo-controlled study**

Binghe Xu^1^*^,†^ • Wei Li^2,†^ • Qingyuan Zhang^3,†^ • Zhimin Shao^4^ • Qiao Li^1^ • Xiaojia Wang^5^ • Huiping Li^6^ • Tao Sun^7^ • Yongmei Yin^8^ • Hong Zheng^9,‡^ • Jifeng Feng^10^ • Hong Zhang^11^ • Guiyuan Lei^12^ • Eleonora Restuccia^13^

^1^ National Cancer Center/National Clinical Research Center for Cancer/Cancer Hospital, Chinese Academy of Medical Sciences, and Peking Union Medical College, Beijing, China ([xubinghe@medmail.com.cn](mailto:xubinghe@medmail.com.cn); [liqiaopumc@qq.com](mailto:liqiaopumc@qq.com))

^2^ The Cancer Center, The First Hospital of Jilin University, Jilin, China ([jdyylw@163.com](mailto:jdyylw@163.com))

^3^ Harbin Medical University, Harbin, China ([zhma19650210@163.com](mailto:zhma19650210@163.com))

^4^ Fudan University Shanghai Cancer Center, Shanghai, China ([zhimingshao@yahoo.com](mailto:zhimingshao@yahoo.com))

^5^ Zhejiang Cancer Hospital, Hangzhou City, China ([wxiaojia0803@163.com](mailto:wxiaojia0803@163.com))

^6^ Beijing Cancer Hospital, Beijing, China ([huipingli2012@hotmail.com](mailto:huipingli2012@hotmail.com))

^7^ Cancer Hospital of China Medical University, Liaoning Cancer Hospital & Institute, Liaoning, China ([jianong@126.com](mailto:jianong@126.com))

^8^ Jiangsu Province Hospital, Nanjing, China ([ym.yin@hotmail.com](mailto:ym.yin@hotmail.com))

^9^ West China Hospital, Sichuan University, Chengdu, China ([hong_zheng11@yahoo.com](mailto:hong_zheng11@yahoo.com))

^10^ Jiangsu Cancer Hospital, Nanjing, China ([fjif@vip.sina.com](mailto:fjif@vip.sina.com))

^11^ Roche (China) Holding Ltd, Shanghai, China ([hong.zhang.hz8@roche.com](mailto:hong.zhang.hz8@roche.com))

^12^ Roche Products Limited, Welwyn Garden City, UK ([guiyuan.lei@roche.com](mailto:guiyuan.lei@roche.com))

^13^ F. Hoffmann-La Roche Ltd, Basel, Switzerland ([eleonora.restuccia@roche.com](mailto:eleonora.restuccia@roche.com)).

* Correspondence:
Professor Binghe Xu
National Cancer Center/National Clinical Research Center for Cancer/Cancer Hospital, Chinese Academy of Medical Sciences, and Peking Union Medical College, Beijing, China
Tel: +8610 87788826

E-mail: [xubinghe@medmail.com.cn](mailto:xubinghe@medmail.com.cn)

^†^ Binghe Xu, Wei Li and Qingyuan Zhang contributed equally to this work.

^‡^ Current affiliation: Janssen China R&D, Beijing, China

**Additional file 1: Figure S1** Patient disposition


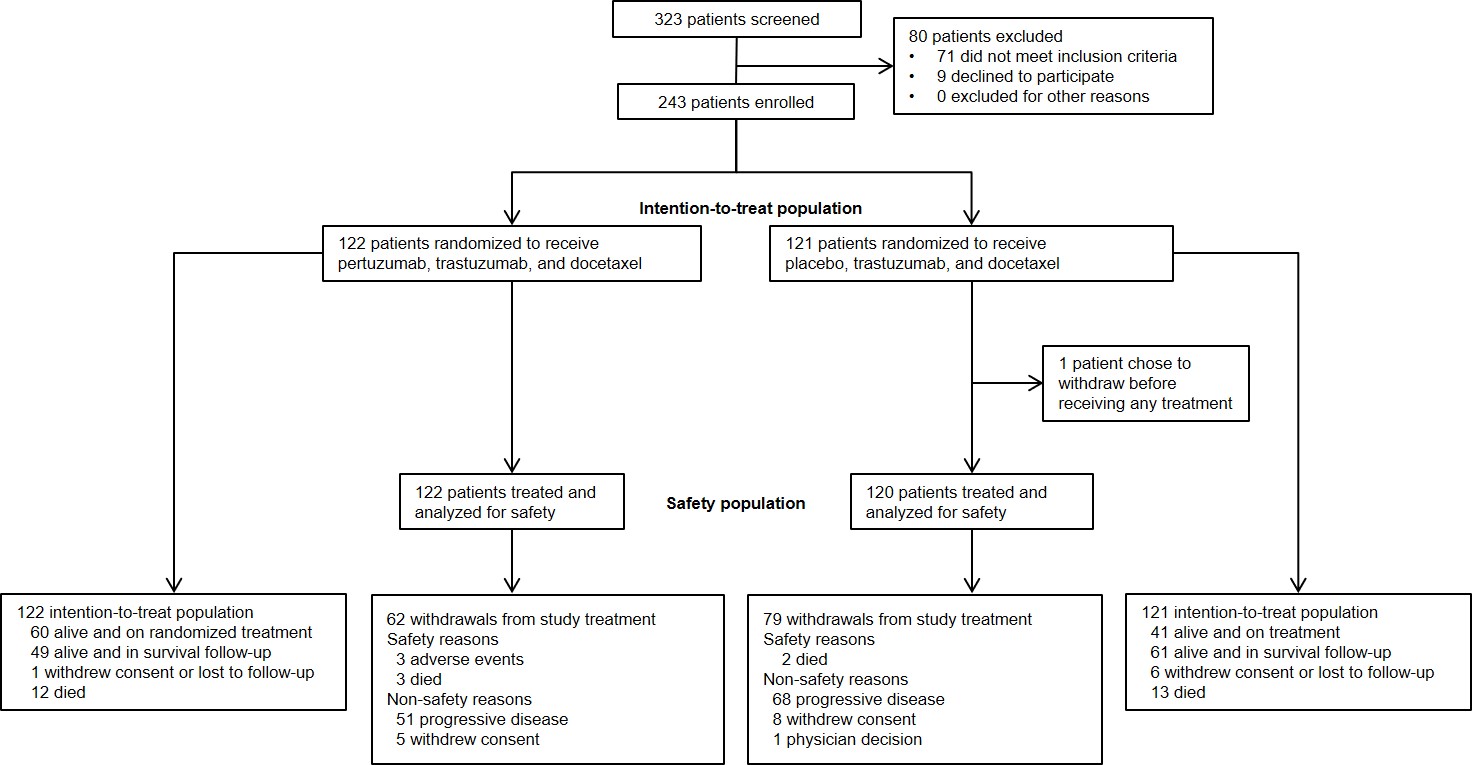


**Additional file 2: Table S1** Exploratory assessment of objective response rate by hormone receptor status in patients with measurable disease at baseline

| Response | Pertuzumab  plus trastuzumab  plus docetaxel | Placebo  plus trastuzumab  plus docetaxel |
| --- | --- | --- |
| Estrogen- and/or progesterone  receptor-positive, *n/N* (%) | 49/60 (81.7) | 43/58 (74.1) |
| 95% CI | 69.6, 90.5 | 61.0, 84.7 |
| Estrogen- and progesterone  receptor-negative, *n/N* (%) | 34/45 (75.6) | 24/39 (61.5) |
| 95% CI | 60.5, 87.1 | 44.6, 76.6 |

Abbreviation: *CI* confidence interval

**Additional file 3: Table S2** Adverse events of interest with pertuzumab therapy

| Patients with at least one event: | Pertuzumab  plus trastuzumab  plus docetaxel  (*n* = 122) | Placebo  plus trastuzumab  plus docetaxel  (*n* = 120) |
| --- | --- | --- |
| Diarrhea | 56 (45.9) | 26 (21.7) |
| Grade ≥3 | 5 (4.1) | 3 (2.5) |
| Rash^a^ | 34 (27.9) | 20 (16.7) |
| Grade ≥3 | 2 (1.6) | 3 (2.5) |
| Leukopenia^a^ | 95 (77.9) | 88 (73.3) |
| Grade ≥3 | 72 (59.0) | 73 (60.8) |
| Leukopenia infection | 5 (4.1) | 3 (2.5) |
| Grade ≥3 | 1 (0.8) | 0 |
| Febrile neutropenia | 5 (4.1) | 7 (5.8) |
| Grade ≥3 | 5 (4.1) | 7 (5.8) |
| Febrile neutropenia infection | 0 | 0 |
| Grade ≥3 | 0 | 0 |
| Anaphylaxis and hypersensitivity | 1 (0.8) | 1 (0.8) |
| Grade ≥3 | 0 | 1 (0.8) |
| Infusion-related reactions | 38 (31.1) | 27 (22.5) |
| Grade ≥3 | 2 (1.6) | 4 (3.3) |
| Mucositis | 27 (22.1) | 12 (10.0) |
| Grade ≥3 | 3 (2.5) | 0 |
| Interstitial lung disease | 2 (1.6) | 4 (3.3) |
| Grade ≥3 | 0 | 0 |

Data are *n* (%)

^a^Events to monitor, i.e., events that the health authorities requested to be monitored closely (usually potential risks or missing information)

**Additional file 4: List of ethics committees**

This study was approved by the following independent ethics committees (IECs):

IEC of Cancer Institute & Hospital, Chinese Academy of Medical Sciences, Beijing, China; IEC of Zhejiang Cancer Hospital, Hangzhou, China; IEC of Fudan University Shanghai Cancer Center, Shanghai, China; IEC of Jiangsu Cancer Hospital, Nanjing, China; IEC of Fuzhou General Hospital, People’s Liberation Army, Nanjing Military Area Command, Fuzhou, China; IEC of Beijing Cancer Hospital, Beijing, China; Local IEC of Changzhou First People’s Hospital, Changzhou, China; IEC of the Cancer Hospital of Harbin Medical University, Haerbin, China; IEC of the First Hospital of Jilin University, Changchun, China; IEC of West China Hospital, Sichuan University, Chengdu, China; IEC of Guangdong General Hospital, Guangzhou, China; IEC of Jiangsu Province People’s Hospital, Nanjing, China; IEC of the First Hospital of China Medical University, Shenyang, China; IEC of General Hospital of People’s Liberation Army, Beijing, China; Liaoning Cancer Hospital & Institute; International IEC of Liaoning Cancer Hospital & Institute, Shenyang, China.

**Additional file 5: Collaborators**

*The following investigators also participated in the PUFFIN study:*

Xuenong Ouyang (Fuzhou General Hospital, People’s Liberation Army, Nanjing Military Area Command, Nanjing), Changping Wu (The First People's Hospital of Changzhou, Changzhou), Ning Liao (Guangdong General Hospital, Guangzhou), Yue-e Teng (The First Hospital of China Medical University, Heping), and Junlan Yang (Chinese People’s Liberation Army General Hospital, Beijing).
